# Temporal Lactate Kinetics and Tissue Lactate Burden in Experimental Skeletal Muscle Ischemia–Reperfusion: Associations with Inflammation, Cellular Injury, and Oxidative Stress

**DOI:** 10.3390/diagnostics16213542

**Published:** 2026-10-28

**Authors:** Alina Belu, Nina Filip, Bogdan Ionel Tamba, Oriana-Maria Onicescu, Gabriela Bordeianu, Alexandru Filip, Alina Jehac, Ștefan Popa, Răzvan Tudor, Rodica Elena Heredea, Andrei Szilagyi, Ivona Mazarianu, Constantin Roman, Elena Cojocaru

**Affiliations:** 1 “Grigore T. Popa” University of Medicine and Pharmacy, 700115 Iasi, Romania; belu_alina@d.umfiasi.ro (A.B.); bogdan.tamba@umfiasi.ro (B.I.T.); gabriela.bordeianu@umfiasi.ro (G.B.); alexandru-filip@umfiasi.ro (A.F.); alina.jehac@umfiasi.ro (A.J.); stefan.popa@umfiasi.ro (Ș.P.); razvan.tudor@umfiasi.ro (R.T.); elena2.cojocaru@umfiasi.ro (E.C.); 2 Advanced Research and Development Center for Experimental Medicine “Prof. Ostin C. Mungiu”—CEMEX, “Grigore T. Popa” University of Medicine and Pharmacy, 700115 Iasi, Romania; andrei.szilagyi@umfiasi.ro (A.S.); ivona.mazarianu@umfiasi.ro (I.M.); roman.constantin@umfiasi.ro (C.R.); 3 Computer Science Faculty, Alexandru Ioan Cuza University of Iasi, 16 General Berthelot Street, 700483 Iasi, Romania; oriana.oniciuc@info.uaic.ro; 4 Department of Clinical Practical Skills, “Victor Babes” University of Medicine and Pharmacy, Eftimie Murgu Square No. 2, 300041 Timisoara, Romania; elena-rodica.heredea@umft.ro; 5 Center of Expertise for Rare Vascular Disease in Children, Louis Turcanu Children Hospital, 300041 Timisoara, Romania; 6 Department of Biology, Faculty of Biology, Alexandru Ioan Cuza University of Iasi, 700506 Iasi, Romania

**Keywords:** skeletal muscle ischemia–reperfusion, lactate kinetics, tissue lactate, oxidative stress, interleukin-6, metabolic recovery

## Abstract

**Background:** Skeletal muscle ischemia–reperfusion (I/R) induces metabolic, inflammatory, and oxidative disturbances that may persist after restoration of blood flow. This study evaluated circulating lactate kinetics together with tissue lactate, inflammatory, cellular injury, and oxidative markers following experimental skeletal muscle I/R. **Methods:** Animals subjected to skeletal muscle I/R were compared with vascular I/R, bone fracture, sham, and control groups. Circulating lactate, PO_2_, glucose, hematocrit, and calculated oxygen saturation were assessed immediately after ischemia, approximately 15 min after reperfusion, and approximately 2 h after reperfusion. Tissue and plasma lactate, lactate dehydrogenase (LDH), interleukin-6 (IL-6), malondialdehyde (MDA), and superoxide dismutase (SOD) were also evaluated. **Results:** In the skeletal muscle I/R group, circulating lactate decreased from 2.19 ± 0.67 mmol/L immediately after ischemia to 1.44 ± 0.84 mmol/L during early reperfusion, before rising to 3.33 ± 1.15 mmol/L at approximately 2 h (Friedman *p* < 0.001), resulting in a negative lactate clearance. This biphasic pattern contrasted with the progressive decline in lactate observed in the vascular I/R and bone fracture groups. Tissue lactate was markedly elevated (11.96 ± 1.27 mmol/L) and showed positive correlations with MDA (ρ = 0.802), IL-6 (ρ = 0.741), and LDH (ρ = 0.671), all *p* < 0.001. **Conclusions:** Skeletal muscle I/R was characterized by a late increase in circulating lactate together with elevated tissue lactate and biochemical evidence of inflammatory activation, cellular injury, and oxidative stress. Serial lactate assessment may provide complementary information on post-ischemic metabolic recovery, although further validation is required.

## 1. Introduction

Skeletal muscle ischemia–reperfusion injury (IRI) is a complex process in which restoration of blood flow to previously ischemic tissue may itself contribute to further cellular damage. The intensity of the injury is related not only to the duration and severity of ischemia, but also to the metabolic and vascular changes that develop during early reperfusion. These include oxidative stress, inflammatory activation, endothelial and microvascular dysfunction, and disturbances in cellular metabolism. Experimental models of lower-limb IRI have shown that reperfusion may induce a systemic inflammatory response, with increased levels of cytokines such as interleukin-6 (IL-6) and may also contribute to injury in distant organs [1]. Subsequent experimental studies have further demonstrated that local tissue damage during reperfusion is closely associated with inflammatory activation and oxidative imbalance [2,3].

Metabolic changes develop early during skeletal muscle ischemia. As oxygen availability decreases, energy production shifts toward anaerobic glycolysis, leading to increased glucose utilization and accumulation of lactate within the affected tissue. Human microdialysis studies have documented dynamic changes in skeletal muscle lactate concentrations during both ischemia and reperfusion [4,5]. In tourniquet-induced ischemia, intramuscular lactate rises markedly and may then gradually decrease after blood flow is restored, under favorable tissue recovery conditions [5]. Similar findings have been reported in experimental models, in which progressive lactate accumulation during ischemia was accompanied by changes in antioxidant defense systems [3,6]. These data indicate that lactate kinetics may offer more information than an isolated measurement, as they reflect both the metabolic load generated during ischemia and the subsequent recovery of tissue metabolism after reperfusion.

Reperfusion activates oxidative and inflammatory pathways that may further aggravate skeletal muscle injury. Experimental studies have consistently shown increased production of reactive oxygen species, lipid peroxidation, and alterations in endogenous antioxidant defenses during skeletal muscle IRI [7]. Large-animal experimental models have also demonstrated concomitant changes in inflammatory mediators such as IL-6, markers of cellular injury including lactate dehydrogenase, and oxidative stress parameters such as malondialdehyde and superoxide dismutase [1,2]. Despite these findings, metabolic, inflammatory, and oxidative changes have generally been examined as separate components of the reperfusion response.

The relationship between circulating lactate kinetics, tissue lactate accumulation, inflammatory activation, and oxidative stress remains incompletely understood in skeletal muscle ischemia–reperfusion injury (IRI). The present study aimed to examine these processes together by combining serial measurements of circulating lactate with tissue lactate concentrations and biochemical markers of cellular injury, inflammation, and oxidative stress.

Our study focused on lactate kinetics following skeletal muscle ischemia–reperfusion (I/R), using vascular I/R and bone injury models for comparison. These complementary experimental models were included to assess whether the systemic lactate response observed after skeletal muscle I/R differed from that associated with other mechanisms of peripheral tissue injury. This comparative approach aimed to better characterize the temporal evolution of lactate following skeletal muscle I/R and its relationship to the underlying mechanism of injury.

## 2. Materials and Methods

### 2.1. Animals and Ethical Approval

Male Wistar rats, 12 weeks of age and weighing approximately 250 g at arrival, were obtained from the Cantacuzino National Medical-Military Institute for Research and Development. After a 2-week quarantine period followed by 2 weeks of acclimatization, the animals were housed in individually ventilated cages under controlled environmental conditions (20 ± 4 °C, relative humidity 50 ± 5%, 12 h light/12 h dark cycle). Standard laboratory chow and water were provided ad libitum. All animals underwent daily clinical observation throughout the experimental period.

All procedures were performed at the Advanced Research and Development Center for Experimental Medicine “Prof. Ostin C. Mungiu” (CEMEX), Grigore T. Popa University of Medicine and Pharmacy, Iași, Romania, in accordance with Directive 2010/63/EU and Romanian Law No. 43/2014. The protocol was approved by the Ethics Committee of Grigore T. Popa University of Medicine and Pharmacy (Approval No. 505/21 December 2024) and by the Romanian National Sanitary Veterinary and Food Safety Authority (Approval No. 80/28 May 2025). The study was conducted and reported in accordance with the ARRIVE 2.0 guidelines for animal research [8].

### 2.2. Experimental Design and Groups

The study included one general control group and three experimental models of peripheral tissue injury: skeletal muscle ischemia–reperfusion, vascular ischemia–reperfusion, and bone fracture. The control group comprised six animals. Each experimental model included 14 animals, of which three were assigned to the corresponding sham subgroup and 11 underwent the model-specific procedure. Sham animals underwent anesthesia, surgical exposure, tissue manipulation, and perioperative handling without induction of the corresponding experimental lesion.

For consistency throughout the manuscript, the experimental groups were coded as follows: M, skeletal muscle ischemia–reperfusion; V, vascular ischemia–reperfusion; O, osseous injury (tibial fracture); C, control; and S, sham.

M group was the primary group of interest in the present analysis. The experimental procedure was based on previously described models of skeletal muscle ischemia–reperfusion in rats [9,10]. The ligation procedure was performed consistently across animals, following the same experimental protocol.

In the V group, the femoral neurovascular bundle was surgically exposed and clamped. This approach was based on previously described experimental models involving femoral vessel occlusion [11]. The clamp was applied to the entire bundle, including the femoral artery, vein, and nerve. After the ischemic period, the clamp was removed to allow reperfusion. The same procedure was performed in all animals.

In the O group, the tibia was surgically exposed, and fracture was induced using bone forceps. Care was taken to minimize damage to the surrounding soft tissues and neurovascular structures. The major blood vessels were not directly manipulated or intentionally injured, and no visible vascular damage or significant bleeding was observed during the procedure [12,13].

Comparisons with the V and O groups were performed to assess whether the metabolic response to skeletal muscle ischemia–reperfusion differed from that observed after vascular occlusion or bone injury. The C group and the corresponding sham group provided baseline and procedural references, respectively. Reporting of the experimental design and animal allocation followed the ARRIVE 2.0 recommendations [14].

### 2.3. Anesthesia and Surgical Procedures

All procedures were performed under general anesthesia. Anesthesia was induced with 5% inhalational isoflurane in an induction chamber and maintained through a nose-cone system using 1.8–2.5% isoflurane in oxygen. Animals were positioned on a thermostatically controlled heated surgical bed to minimize perioperative hypothermia. Perioperative analgesia was provided by intraperitoneal tramadol at approximately 33.3 mg/kg.

Experimental skeletal muscle ischemia was induced in the right hindlimb using a ligation-based procedure, followed by reperfusion. After clipping the hair, the surgical field was prepared under aseptic conditions using alcohol followed by povidone–iodine solution. A skin incision was made with a No. 10 scalpel blade to expose the medial gastrocnemius (MG) muscle. Approximately 20% of the exposed muscle mass was ligated using 3/0 polypropylene non-absorbable suture material to induce localized muscle ischemia. The skin incision was then closed with surgical adhesive (Surgibond^®^).

The corresponding skeletal muscle sham procedure reproduced anesthesia, surgical exposure of the medial gastrocnemius, tissue manipulation, and perioperative handling, but no muscle ligation was performed and ischemia was not induced.

### 2.4. Blood and Tissue Sampling and Biochemical Analyses

Blood samples were collected by retro-orbital sinus puncture. Reperfusion was subsequently initiated by restoring local tissue perfusion. Animals were monitored throughout the postoperative period and until complete recovery from anesthesia, with particular attention to locomotor activity, surgical-site integrity, and the occurrence of postoperative complications.

Blood gas and metabolic parameters were assessed at three predefined time points to capture distinct phases of the metabolic response: T1, immediately after the ischemic period; T2, approximately 15 min after reperfusion; and T3, approximately 2 h after reperfusion. Reperfusion was maintained for approximately 2 h after restoration of blood flow. The 2 h interval was selected to provide a later post-reperfusion assessment clearly separated from the early 15 min measurement and to evaluate whether the metabolic response persisted beyond the immediate reperfusion phase. Previous experimental studies have assessed skeletal muscle ischemia–reperfusion responses over sequential reperfusion periods extending from the first minutes to several hours measurements to characterize evolving metabolic and tissue responses [3,15,16]. This temporal design allowed evaluation of whether circulating lactate changes were transient or persisted beyond the early recovery phase. Measurements included circulating lactate, partial pressure of oxygen (PO_2_), glucose, hematocrit, and calculated oxygen saturation (SO_2_c). Blood gas and metabolic measurements were performed using a GEM Premier 3500 analyzer according to the manufacturer’s instructions (Instrumentation Laboratory, Bedford, MA, USA). The same sampling and measurement procedures were applied across all groups to minimize pre-analytical variability. Samples with insufficient volume or technical measurement errors were excluded from the analysis.

At the end of the experimental protocol (T3, approximately 2 h after reperfusion), plasma and tissue samples were collected for biochemical analysis, separately from the serial blood gas and metabolic measurements performed at T1, T2, and T3. Biochemical analyses included plasma and tissue lactate, plasma lactate dehydrogenase (LDH), interleukin-6 (IL-6), malondialdehyde (MDA), superoxide dismutase (SOD), and total protein concentration. These measurements were used to assess metabolic alterations, cellular injury, inflammatory activation, lipid peroxidation, and antioxidant responses associated with skeletal muscle ischemia–reperfusion.

### 2.5. Lactate and Redox Parameters

The temporal lactate response following ischemia–reperfusion phase was characterized using serial circulating lactate concentrations, peak lactate, final lactate, temporal slope, and calculated lactate clearance. Lactate clearance was calculated according to the formula:

Lactate clearance (%) = [(initial lactate − final lactate)/initial lactate] × 100.

A positive value indicated a decrease in lactate concentration over time, whereas a negative value indicated a net increase during the observation period.

Oxidative stress was assessed using superoxide dismutase (SOD) activity and malondialdehyde (MDA) levels. These parameters were analyzed individually to characterize antioxidant defense and lipid peroxidation during reperfusion.

### 2.6. Statistical Analysis

Data distribution was assessed using the Shapiro–Wilk test. Because deviations from normality were observed for several variables, non-parametric methods were used for inferential analyses. Longitudinal changes across T1, T2, and T3 were evaluated using the Friedman test, with paired T1–T3 comparisons performed using the Wilcoxon signed-rank test. Between-group differences were assessed using the Kruskal–Wallis test, followed, where appropriate, by pairwise Mann–Whitney U tests. Adjustment for multiple pairwise comparisons was performed using the Bonferroni correction.

Associations between metabolic, inflammatory, cellular injury, and oxidative–redox parameters were assessed using Spearman’s rank correlation coefficient (ρ). All tests were two-sided, and a *p* value < 0.05 was considered statistically significant. For post hoc pairwise comparisons, statistical significance was evaluated after Bonferroni adjustment.

Statistical analyses were performed in Python 3.8 (Python Software Foundation, Wilmington, DE, USA) using the SciPy 1.10 and Statsmodels 0.14.

## 3. Results

Before comparing the three experimental injury models, the systemic lactate response associated with each intervention was first evaluated relative to its corresponding sham condition. This analysis was performed to distinguish intervention-related metabolic changes from temporal and procedural variations associated with anesthesia, surgical manipulation, and repeated blood sampling.

### 3.1. Comparison of Each Experimental Model with Its Corresponding Sham Group

#### 3.1.1. Skeletal Muscle Ischemia–Reperfusion Versus Sham

**Figure 1:**
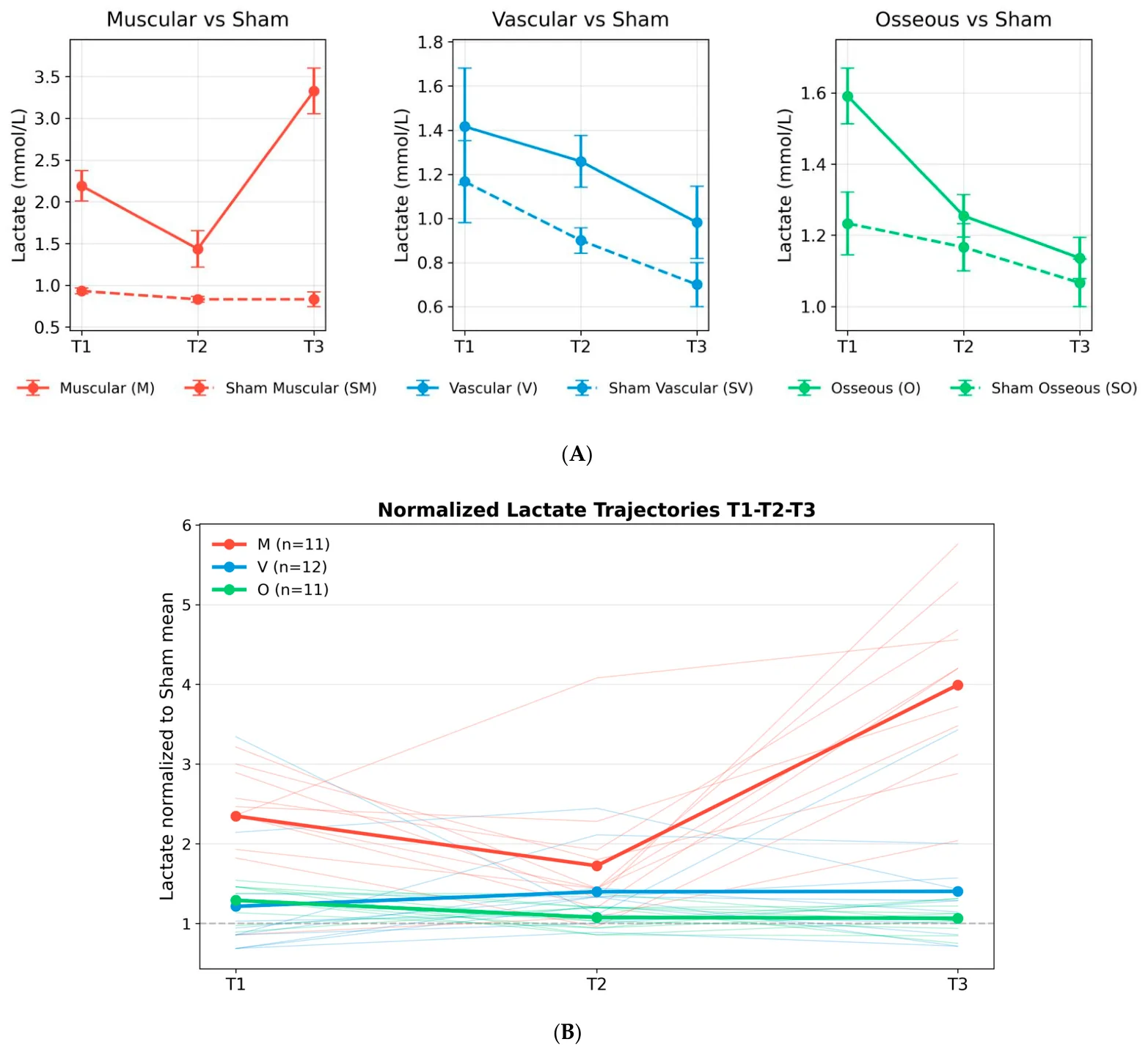
(**A**) Circulating lactate concentrations in the experimental models and their corresponding sham groups. Lactate concentrations at T1, T2, and T3 were compared between each experimental group and its corresponding sham group: M, skeletal muscle ischemia–reperfusion group; V, vascular ischemia–reperfusion group; O, osseous injury group; S, corresponding sham-operated groups. Between-group comparisons were performed using the Mann–Whitney U test. (**B**) Longitudinal trajectories of circulating lactate concentrations normalized to sham across T1, T2, and T3 in the experimental groups. Experimental time points: T1, end of ischemia; T2, approximately 15 min after the initiation of reperfusion; T3, approximately 2 h after the initiation of reperfusion.

Circulating lactate concentrations were higher in the skeletal muscle ischemia–reperfusion group than in the corresponding sham group at all three sampling points. Values were 2.19 versus 0.93 mmol/L at T1 (*p* = 0.042), 1.44 versus 0.83 mmol/L at T2 (*p* = 0.034), and 3.33 versus 0.83 mmol/L at T3 (*p* = 0.013) (Figure 1A).

Lactate concentrations for individual subjects (thin lines) and groups mean trajectories (thick lines with markers) are shown for group M (red), group V (blue), and group O (green). All values are normalized relative to the Sham mean baseline (indicated by the dashed horizontal line at y = 1). Group M demonstrates a marked increase in relative lactate levels by T3 compared to groups V and O, which remain stable near baseline levels throughout the observed period (Figure 1B).

#### 3.1.2. Vascular Ischemia–Reperfusion Versus Sham

Mean circulating lactate concentrations in the vascular ischemia–reperfusion group were approximately 1.41, 1.26, and 0.98 mmol/L at T1, T2, and T3, respectively, compared with 1.17, 0.90, and 0.70 mmol/L in the corresponding sham group. Lactate concentrations decreased from T1 to T3 in both groups (Figure 1A).

#### 3.1.3. Bone Fracture Versus Sham

Mean circulating lactate concentrations in the bone fracture group were approximately 1.59, 1.26, and 1.14 mmol/L at T1, T2, and T3, respectively, compared with 1.23, 1.17, and 1.07 mmol/L in the corresponding sham group. Lactate concentrations decreased in both groups, and the difference between their means narrowed over time (Figure 1A).

In addition, at T3, PO_2_ was also higher in the skeletal muscle ischemia–reperfusion group than in the corresponding sham group (179.64 vs. 88.00 mmHg, *p* = 0.013). Glucose was 142.64 versus 113.00 mg/dL (*p* = 0.039), while calculated oxygen saturation was 99.91% versus 99.00% (*p* = 0.039).

### 3.2. Comparative Systemic Lactate Dynamics Among Experimental Injury Models

The skeletal muscle ischemia–reperfusion group showed a distinct temporal lactate pattern across the three sampling points. Circulating lactate was 2.19 ± 0.67 mmol/L at T1, decreased to 1.44 ± 0.84 mmol/L at T2, and subsequently increased to 3.33 ± 1.15 mmol/L at T3. The overall change across time was statistically significant (Friedman χ^2^ = 14.73, *p* < 0.001).

The mean peak lactate concentration in the skeletal muscle I/R group was 3.37 ± 0.87 mmol/L, while the final lactate concentration was 3.33 ± 1.15 mmol/L. Calculated lactate clearance was negative (−59.0 ± 40.3%), and the mean temporal lactate slope was positive (+0.57 ± 0.46 mmol/L per sampling interval).

**Figure 2:**
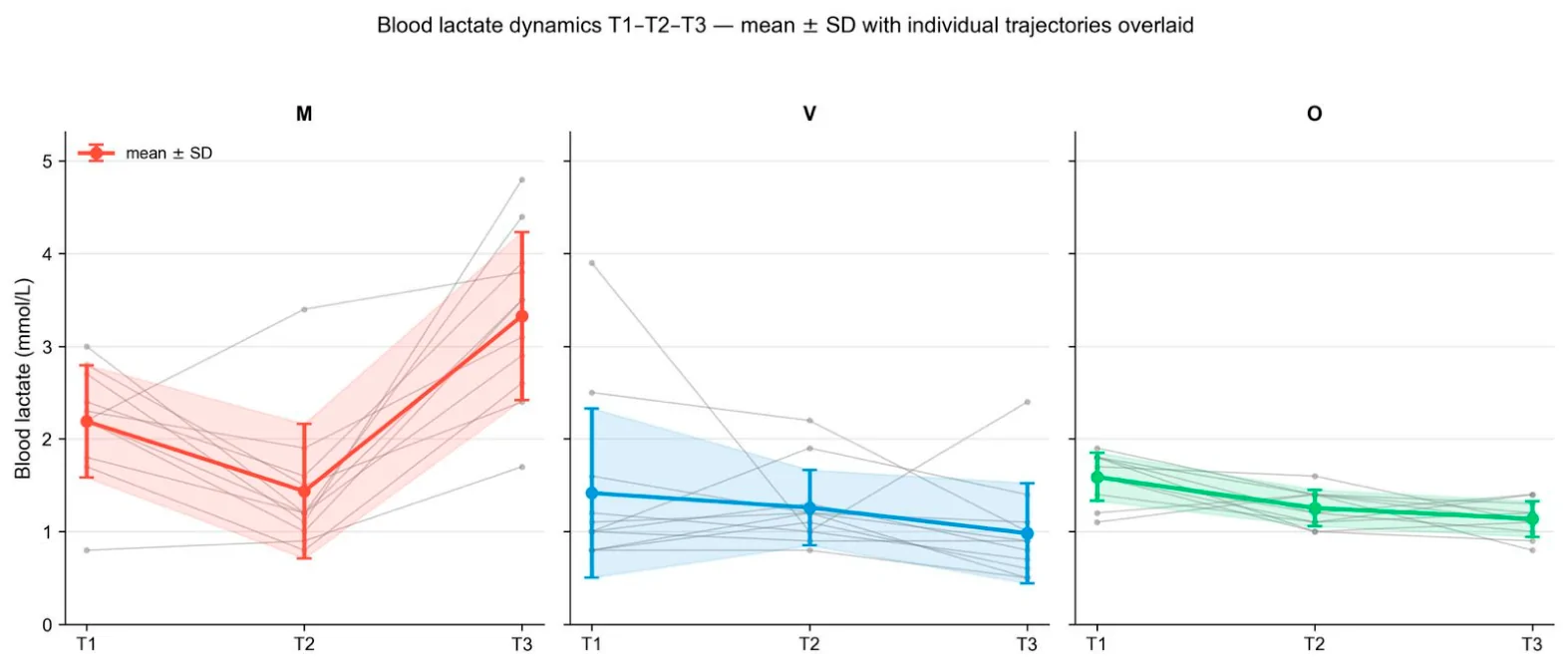
Temporal dynamics of circulating lactate in the experimental models. Group-level and individual lactate trajectories at T1, T2, and T3. The overall temporal change in the skeletal muscle ischemia–reperfusion group was significant (Friedman χ^2^ = 14.73, *p* < 0.001). M, skeletal muscle ischemia–reperfusion group; V, vascular ischemia–reperfusion group; O, osseous injury group; T1, immediately after ischemia; T2, approximately 15 min after reperfusion; T3, approximately 2 h after the initiation of reperfusion.

By comparison, the vascular I/R and bone fracture groups showed lower final lactate concentrations and positive lactate clearance. Final lactate concentrations were 0.98 ± 0.56 mmol/L in the vascular I/R group and 1.14 ± 0.18 mmol/L in the bone fracture group, with calculated lactate clearances of +22.8 ± 27.0% and +27.6 ± 12.5%, respectively (Figure 2).

### 3.3. Comparative Tissue and Plasma Biochemical Profile

The skeletal muscle ischemia–reperfusion group showed higher plasma and tissue lactate concentrations than the vascular I/R and control groups. In the terminal biochemical samples, plasma lactate was 6.84 ± 0.20 mmol/L in the skeletal muscle I/R group, compared with 3.94 ± 0.63 mmol/L in the vascular I/R group (*p* < 0.001) and 5.87 ± 0.46 mmol/L in the control group (*p* = 0.008). The late value refers to plasma lactate measured in terminal biochemical samples from the general control group using a separate biochemical assay.

Tissue lactate was 11.96 ± 1.27 mmol/L in the skeletal muscle I/R group, compared with 8.89 ± 0.73 mmol/L in the vascular I/R group and 6.82 ± 0.55 mmol/L in the control group (both *p* < 0.001).

LDH was higher in the skeletal muscle I/R group than in both the vascular I/R and control groups (297.82 ± 12.53 vs. 244.55 ± 20.24 and 254.17 ± 7.58 U/L; *p* < 0.001 and *p* = 0.003, respectively). IL-6 showed a similar pattern, with values of 17.43 ± 2.90 pg/mL in the skeletal muscle I/R group, 12.45 ± 1.35 pg/mL in the vascular I/R group, and 12.30 ± 0.44 pg/mL in the control group (both comparisons *p* < 0.001).

**Figure 3:**
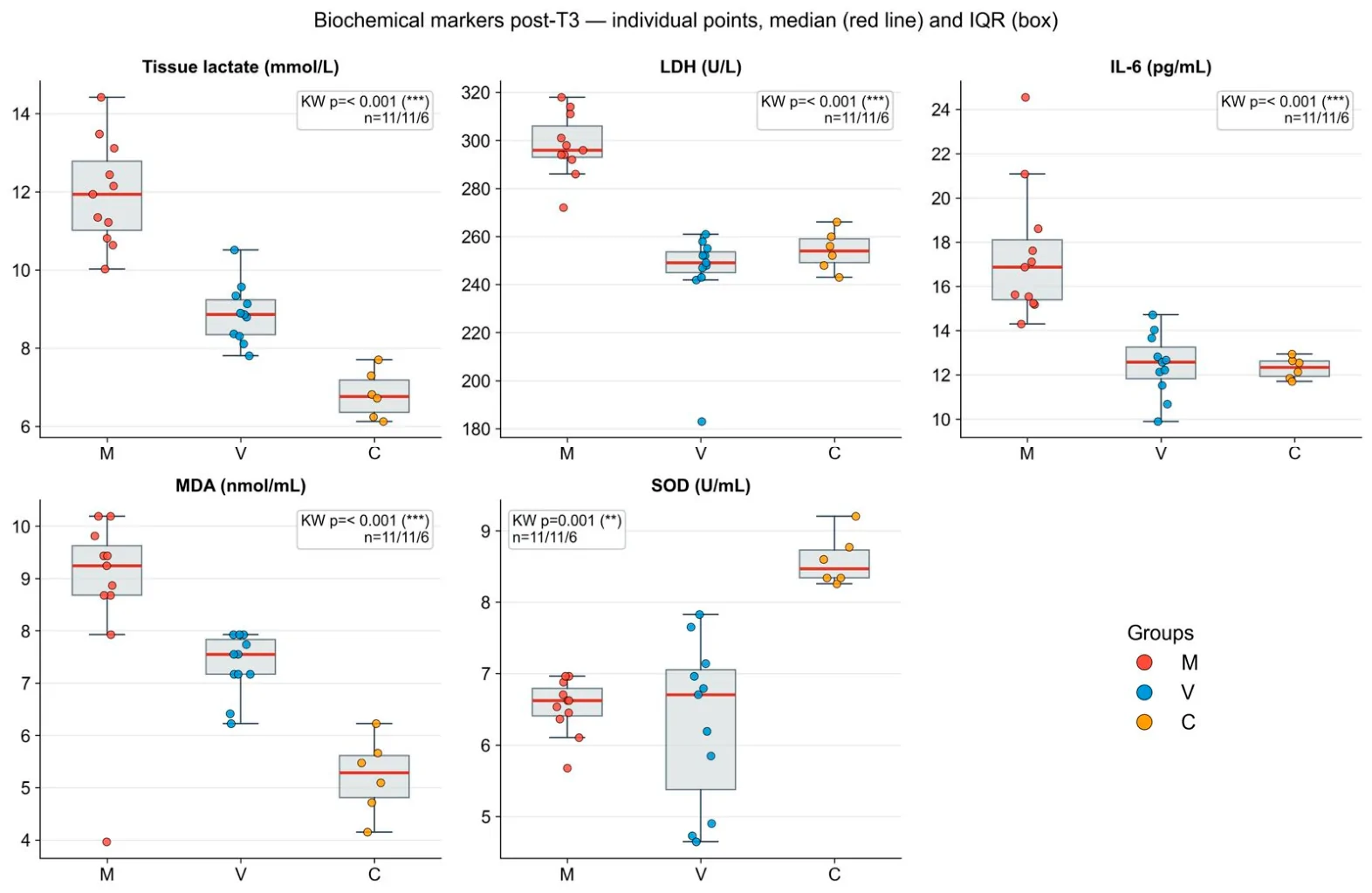
Comparison of biochemical profiles among the skeletal muscle ischemia–reperfusion, vascular ischemia–reperfusion, and control groups. Comparison of tissue lactate, plasma lactate dehydrogenase (LDH), interleukin-6 (IL-6), malondialdehyde (MDA), and superoxide dismutase (SOD) among the experimental groups. Data are displayed as box plots showing medians and interquartile ranges (IQRs). M, skeletal muscle ischemia–reperfusion group; V, vascular ischemia–reperfusion group; C, control group. Statistical significance: ** *p* < 0.01; *** *p* < 0.001.

MDA was also higher in the skeletal muscle I/R group (8.77 ± 1.65 nmol/mL) than in the vascular I/R group (7.34 ± 0.56 nmol/mL, *p* = 0.010) and the control group (5.22 ± 0.67 nmol/mL, *p* = 0.046). Total protein concentration was lower in the skeletal muscle I/R group (5.37 ± 0.60 g/dL) than in the vascular I/R group (6.52 ± 0.44 g/dL, *p* = 0.003) and the control group (7.13 ± 0.78 g/dL, *p* = 0.004) (Figure 3).

### 3.4. Oxidative Stress Profile

Compared with the general control group, the skeletal muscle ischemia–reperfusion group showed lower SOD activity (6.54 ± 0.37 vs. 8.59 ± 0.33 U/mL, *p* = 0.006).

### 3.5. Associations Between Tissue Lactate and Biochemical Markers

Tissue lactate showed significant positive correlations with markers of tissue injury, inflammation, and oxidative stress. Tissue lactate correlated with LDH (Spearman ρ = 0.671, *p* < 0.001), IL-6 (ρ = 0.741, *p* < 0.001), and MDA (ρ = 0.802, *p* < 0.001).

**Figure 4:**
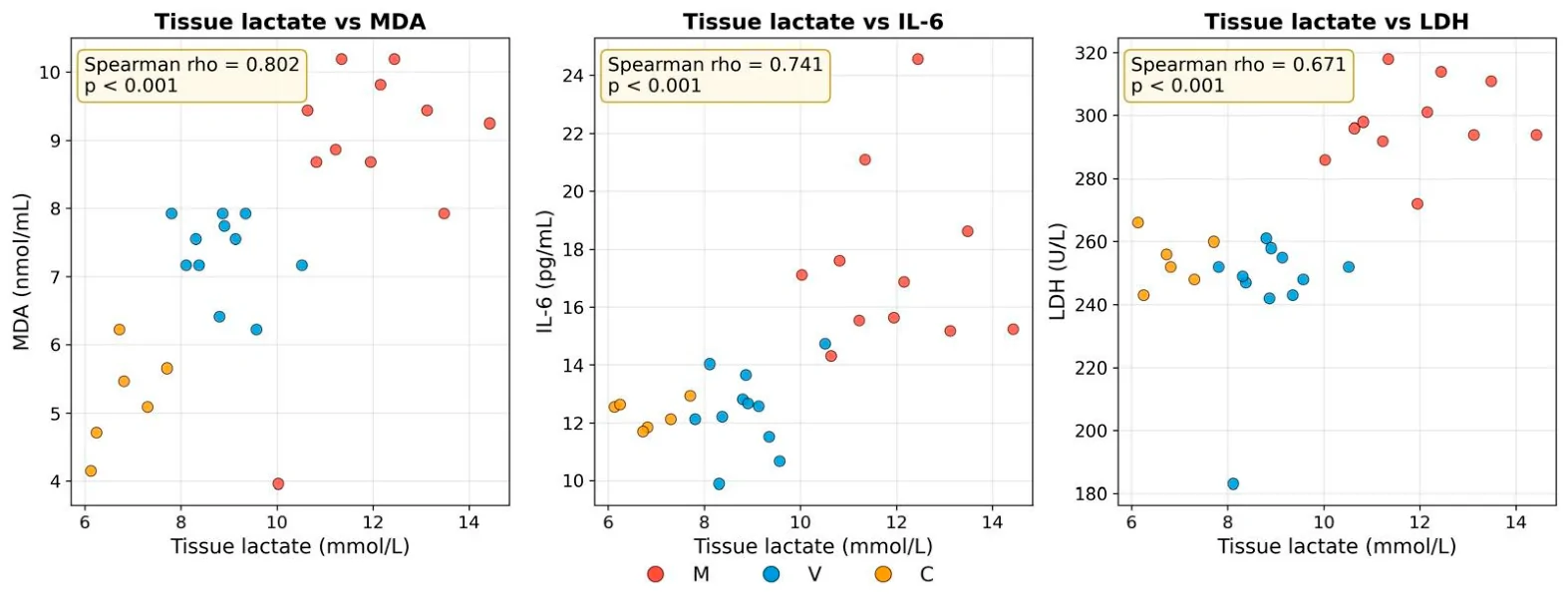
Associations between tissue lactate and biochemical markers in experimental groups. Relationships between tissue lactate and plasma malondialdehyde (MDA), interleukin-6 (IL-6), and lactate dehydrogenase (LDH). Correlations were assessed using Spearman’s rank correlation coefficient. M, skeletal muscle ischemia–reperfusion group; V, vascular ischemia–reperfusion group; C, control group.

A strong inverse correlation was observed between tissue lactate and total protein concentration (ρ = −0.905, *p* < 0.001). Additional significant correlations included LDH with IL-6 (ρ = 0.747, *p* < 0.001), MDA with SOD (ρ = −0.636, *p* < 0.001), total protein concentration with LDH (ρ = −0.686, *p* < 0.001), and total protein concentration with IL-6 (ρ = −0.663, *p* < 0.001) (Figure 4).

## 4. Discussion

Before comparing the temporal lactate responses across the three experimental models, each injury group was evaluated against its corresponding sham condition. Among the three models, statistically significant differences in circulating lactate concentrations were observed only in the skeletal muscle ischemia–reperfusion (I/R) group, with higher values than in sham animals at all three sampling points. Although isoflurane anesthesia may influence lactate metabolism, circulating lactate concentrations remained relatively stable in sham-operated animals exposed to the same anesthetic conditions. These findings support an association between the observed metabolic response and the induced skeletal muscle injury, beyond the effects of anesthesia, surgical exposure, tissue manipulation, and repeated sampling.

The principal finding of this study was the distinct temporal pattern of circulating lactate following skeletal muscle I/R. Lactate concentrations decreased during early reperfusion, between T1 and T2, but subsequently increased to their highest values at T3, resulting in negative calculated clearance and a positive temporal slope. In contrast, the vascular I/R and bone fracture groups showed a progressive decline in lactate concentrations and positive calculated clearance. These differences may partly reflect the extent and metabolic characteristics of the tissues affected by each intervention. Variations in muscle mass and fiber-type composition could influence lactate production and release; however, these characteristics were not independently quantified in the present study. Differences in tissue involvement, ischemia severity, and reperfusion conditions may influence the metabolic responses observed across experimental models [15,16,17]. The observed lactate profiles should therefore be interpreted within the conditions of the present protocol rather than as evidence of a universal hierarchy of injury severity.

The decrease in lactate during early reperfusion followed by a later rise highlights the importance of assessing its course at different stages. Our sampling points were selected on the basis of previous experimental studies examining metabolic changes during distinct phases of reperfusion [3,18,19].

Metabolic disturbances after skeletal muscle ischemia are well established; however, their evolution during reperfusion depends on the duration and severity of ischemia and on the extent of tissue recovery. Korei et al. reported marked metabolic alterations in skeletal muscle after prolonged acute ischemia followed by reperfusion [20], whereas microdialysis studies of tourniquet-induced ischemia demonstrated a rapid increase in muscle lactate followed by gradual normalization after restoration of blood flow [5,21,22]. Taken together, these observations support the potential value of serial lactate assessment, rather than reliance on a single measurement, for characterizing the evolving metabolic response of ischemic skeletal muscle during reperfusion [23,24].

The subsequent increase in lactate during reperfusion is of particular interest. Reperfusion may facilitate the release of metabolites accumulated within ischemic tissue into the circulation, while a delayed increase in circulating lactate may also reflect incomplete metabolic recovery. In patients undergoing distal revascularization for critical limb ischemia, Alves et al. measured lactate concentrations and lipid peroxidation markers in blood and skeletal muscle at successive time points during ischemia and reperfusion, demonstrating parallel temporal changes in these metabolic and oxidative stress parameters [15]. Knee replacement has also been used as a controlled human model of skeletal muscle ischemia–reperfusion, demonstrating marked intramuscular lactate accumulation during ischemia and reperfusion [23]. Experimental hypoxia studies further suggest that normalization of extracellular lactate after restoration of oxygen availability is not immediate [24]. In the present model, elevated tissue lactate, together with the delayed increase in circulating lactate, is consistent with persistent metabolic disturbances beyond the early reperfusion phase, although the underlying cellular mechanisms remain undetermined.

The metabolic response was also accompanied by evidence of inflammatory activation. IL-6 concentrations were higher in the skeletal muscle ischemia–reperfusion group than in both the vascular ischemia–reperfusion and control groups, and tissue lactate showed a positive correlation with IL-6. Previous investigations have documented concurrent inflammatory and redox alterations in human skeletal muscle during ischemia–reperfusion [25], as well as cytokine release following reperfusion in tourniquet-based clinical models [16]. Experimental studies further support inflammation as an important component of skeletal muscle I/R injury, with attenuation of the inflammatory response being associated with reduced tissue damage [17]. These responses may also be influenced by the local muscle environment, including resident inflammatory cells and muscle fiber characteristics [4,26]. Although the positive correlation between tissue lactate and IL-6 indicates that greater metabolic disturbance was accompanied by a stronger inflammatory response, it does not establish a direct causal relationship between lactate accumulation and cytokine signaling.

Oxidative stress was also evident in the skeletal muscle ischemia–reperfusion group, which showed increased MDA levels together with lower SOD activity compared with controls. Direct measurements in reperfused skeletal muscle have demonstrated free-radical production during the reperfusion phase [7,18], supporting the presence of oxidative injury beyond changes inferred from downstream biochemical markers. Increased lipid peroxidation and alterations in endogenous antioxidant defenses have also been reported in several experimental models of skeletal muscle I/R. Wang et al. described changes in MDA and SOD following experimental ischemia–reperfusion [27], while Tong et al. evaluated MDA, SOD, and LDH within the same experimental setting [28]. Studies of ischemic preconditioning have further shown that modifying reperfusion conditions may attenuate both biochemical and histological features of oxidative injury [29]. More recent experimental approaches targeting oxidative stress have likewise demonstrated concurrent changes in metabolic, cellular injury, and prooxidant/antioxidant parameters [30]. Of particular interest was the relationship between tissue lactate and the biochemical markers measured at T3, approximately 2 h after the initiation of reperfusion. Tissue lactate showed positive correlations with MDA (ρ = 0.802), IL-6 (ρ = 0.741), and LDH (ρ = 0.671), with the strongest association observed for MDA. This finding indicates that greater local lactate accumulation occurred alongside more pronounced lipid peroxidation. Previous studies have addressed individual components of this relationship: lactate and oxidative stress markers have been examined concurrently in human skeletal muscle [21,22], while experimental models have evaluated LDH together with MDA and SOD [27,28,30]. However, the combined assessment of serial circulating lactate dynamics, tissue lactate accumulation, inflammatory activation, and oxidative stress within a single experimental model has received comparatively limited attention. By examining these parameters together, the present study provides an integrated characterization of the metabolic, inflammatory, and oxidative responses associated with skeletal muscle reperfusion.

LDH provided additional biochemical evidence of tissue injury. Increased circulating LDH has long been used as a marker of skeletal muscle damage in experimental I/R models, although it is not specific to a particular mechanism of injury. Early murine studies demonstrated differences in both biochemical and histological susceptibility to skeletal muscle ischemia–reperfusion [31], while more recent methodological work has combined circulating injury markers with histological and functional assessment to characterize the time course of lower-limb I/R damage [16]. The consequences of I/R may also extend beyond the early biochemical phase, as impaired structural recovery and persistent muscle dysfunction have been reported after experimental reperfusion injury [32]. The positive association between tissue lactate and LDH observed in our study supports a relationship between metabolic disturbance and biochemical evidence of cellular injury, although the extent of structural muscle damage could not be directly assessed.

A further finding was the marked increase in PO_2_ at T3 despite the concomitant rise in lactate. This apparent dissociation is of particular interest, as restoration of blood oxygen availability does not necessarily imply homogeneous microvascular perfusion or adequate cellular oxygen utilization. Experimental studies have documented increased microvascular permeability and altered vascular function after skeletal muscle ischemia–reperfusion [33]. However, microvascular flow, tissue oxygen extraction, mitochondrial respiration, and regional perfusion heterogeneity were not directly assessed in the present study. Therefore, the observed PO_2_–lactate pattern should not be interpreted as evidence of a demonstrated microcirculatory shunt, but rather as an apparent dissociation between blood oxygen availability and metabolic recovery that permits further physiological investigation.

Several limitations should be considered when interpreting these findings. The sample size was modest, particularly in the sham subgroups, which were intended primarily as procedural reference groups rather than fully powered comparison cohorts. Multiple biochemical parameters and exploratory correlations were evaluated; therefore, these findings should be interpreted with careful consideration, despite adjustment for multiple comparisons where applicable.

Although the experimental intervention was performed according to a standardized protocol and plasma LDH was assessed, myoglobin and creatine kinase MM isoenzyme (CK-MM) concentrations were not determined. These additional biomarkers could have provided further biochemical confirmation of skeletal muscle injury and contributed to a more comprehensive characterization of its extent.

Furthermore, biochemical monitoring was limited to predefined experimental time points, with the final assessment performed approximately 2 h after the initiation of reperfusion. No additional blood or tissue samples were collected beyond this interval, preventing evaluation of later metabolic, inflammatory, and oxidative changes.

In addition, the study did not include direct measurements of mitochondrial respiration, microvascular flow, or oxygen extraction, limiting pathophysiological interpretation of the delayed increase in circulating lactate. Finally, biochemical evidence of tissue injury was not complemented by quantitative histological assessment of muscle damage, an approach that has contributed to a more detailed characterization of I/R severity in previous experimental models [16,32].

Within these limitations, the findings indicate a distinct metabolic pattern following skeletal muscle ischemia–reperfusion. The late rise in circulating lactate occurred together with increased tissue lactate, inflammatory activation, biochemical evidence of tissue injury, and oxidative imbalance. Tissue lactate was also positively associated with IL-6, LDH, and MDA. These observations suggest that lactate should not be interpreted solely as an isolated end product of anaerobic metabolism, as its temporal evolution may provide additional information about the metabolic state of reperfused skeletal muscle. Further studies combining serial lactate measurements with direct assessment of tissue perfusion, oxygen extraction, mitochondrial function, and histological injury are needed to determine whether this integrated pattern can reliably identify incomplete metabolic recovery after reperfusion.

From a translational perspective, these findings suggest the potential value of serial lactate measurements when evaluating metabolic recovery following acute ischemia–reperfusion. A persistent or secondary increase in circulating lactate could prompt closer reassessment of tissue perfusion and recovery, although the present experimental data do not establish clinical thresholds and require validation in human studies. Moreover, these experimental models do not reproduce the full complexity or metabolic magnitude of human disease states, and the findings should not be directly extrapolated to chronic ischemia, which involves different adaptive and metabolic processes. Their relevance therefore lies primarily in characterizing pathophysiological responses to distinct mechanisms of peripheral tissue injury rather than establishing direct clinical equivalence.

## 5. Conclusions

Experimental skeletal muscle ischemia–reperfusion was characterized by a distinct temporal lactate pattern, with a late increase in circulating lactate during reperfusion and elevated tissue lactate concentrations. This metabolic response occurred in parallel with increased IL-6 and LDH levels and biochemical evidence of oxidative stress.

Tissue lactate was positively associated with IL-6, LDH, and MDA, indicating that greater local lactate accumulation was associated with more pronounced inflammatory activation, cellular injury, and lipid peroxidation. These findings support the interpretation of lactate kinetics as part of a broader metabolic response to skeletal muscle ischemia–reperfusion rather than as an isolated biochemical change.

Further studies integrating serial lactate measurements with direct assessments of tissue perfusion, oxygen extraction, mitochondrial function, and histological injury are needed to clarify the mechanisms underlying the late increase in circulating lactate and to determine the reproducibility and translational relevance of this integrated biochemical profile. From a clinical perspective, serial lactate monitoring may warrant further investigation as an adjunct for assessing metabolic recovery after ischemia–reperfusion; however, direct application to patient management requires validation in human studies.

## Data Availability

The data supporting the findings of this study are available from the corresponding author upon reasonable request.

## Abbreviations

The following abbreviations are used in this manuscript:

I/R	Ischemia–reperfusion
IRI	Ischemia–reperfusion injury
IL-6	Interleukin-6
LDH	Lactate dehydrogenase
MDA	Malondialdehyde
SOD	Superoxide dismutase
PO_2_	Partial pressure of oxygen
SO_2_c	Calculated oxygen saturation
MG	Medial gastrocnemius
M	Skeletal muscle ischemia–reperfusion group
A	Vascular ischemia–reperfusion group
O	Bone fracture group
C	Control group
S	Sham group
T1, T2, T3	Predefined sampling time points
